# History of El Niño impacts on the global carbon cycle 1957–2017: a quantification from atmospheric CO_2_ data

**DOI:** 10.1098/rstb.2017.0303

**Published:** 2018-10-08

**Authors:** C. Rödenbeck, S. Zaehle, R. Keeling, M. Heimann

**Affiliations:** 1 Max Planck Institute for Biogeochemistry, Jena, Germany; 2 Scripps Institution of Oceanography, University of California, San Diego, CA, USA; 3 Institute for Atmospheric and Earth System Research (INAR), Faculty of Science, University of Helsinki, Helsinki, Finland

**Keywords:** El Niño, atmospheric CO_2_ data, climate sensitivity

## Abstract

Interannual variations in the large-scale net ecosystem exchange (NEE) of CO_2_ between the terrestrial biosphere and the atmosphere were estimated for 1957–2017 from sustained measurements of atmospheric CO_2_ mixing ratios. As the observations are sparse in the early decades, available records were combined into a ‘quasi-homogeneous’ dataset based on similarity in their signals, to minimize spurious variations from beginning or ending data records. During El Niño events, CO_2_ is anomalously released from the tropical band, and a few months later also in the northern extratropical band. This behaviour can approximately be represented by a linear relationship of the NEE anomalies and local air temperature anomalies, with sensitivity coefficients depending on geographical location and season. The apparent climate sensitivity of global total NEE against variations in pan-tropically averaged annual air temperature slowly changed over time during the 1957–2017 period, first increasing (though less strongly than in previous studies) but then decreasing again. However, only part of this change can be attributed to actual changes in local physiological or ecosystem processes, the rest probably arising from shifts in the geographical area of dominating temperature variations.

This article is part of a discussion meeting issue ‘The impact of the 2015/2016 El Niño on the terrestrial tropical carbon cycle: patterns, mechanisms and implications’.

## 1. Introduction

The El Niño Southern Oscillation (ENSO) is the largest mode of interannual variability both in the climate system [1] and in the global carbon cycle [2]. While both oceanic [3] and terrestrial [4] carbon cycle processes respond to ENSO, the atmospheric CO_2_ variability is dominated by ENSO-related interannual variations of the terrestrial net ecosystem exchange (NEE) of CO_2_ [5]. NEE is understood here as the entire CO_2_ exchange between land ecosystems and the atmosphere, including fires. Climate anomalies cause NEE anomalies through enhancement or suppression of photosynthesis, autotrophic and heterotrophic respiration, biomass burning, and mortality [4]. These same processes also contribute to centennial NEE trends in a changing climate, which can feed back to the climate trends. A quantitative understanding of the climate effects on NEE, including possible decadal or centennial changes, is therefore a necessary condition for realistic climate prediction. In this special issue, ENSO-related variability is employed as a ‘natural experiment’, using the well-observed 2015/2016 El Niño as a study case. The aim of our contribution is to put the estimates of the NEE response during the 2015/2015 El Niño into a historical context, in order to distinguish typical from specific behaviour and to detect possible slow trends in the NEE responses.

For this aim, an analysis period as long as possible is necessary. The longest available observations of carbon cycle variability are sustained atmospheric CO_2_ measurements, started by Keeling [6] at La Jolla Pier (California) and at the South Pole in 1957 and at Mauna Loa (Hawaii) in 1958. Interannual variations of the CO_2_ growth rate in any of these records, approximately reflecting global total CO_2_ flux variations, are clearly ENSO-related [7].

In order to disentangle the contributions of different geographical areas to the variability in atmospheric CO_2_ records, inversion techniques have been applied [5,8–11, and others], bringing to bear the additional information from the spatial gradients available in a set of measurement stations, and from an atmospheric tracer transport model quantitatively linking changes in atmospheric CO_2_ to the underlying CO_2_ exchanges at specific locations and times (instead of assuming instantaneous mixing throughout the atmosphere). However, while there are more than 100 stations globally that regularly measure atmospheric CO_2_ mixing ratios today, only a few of them were in operation before 1980, and even fewer in the 1960s and early 1970s. Owing to this, available inverse estimates of NEE variations do not start much before the 1980s. The first aim of this study, therefore, is to quantify the interannual NEE anomalies on large spatial scales over the full 1957–2017 time period since the start of atmospheric CO_2_ measurements, applying the inversion technique on as many early data as possible, and to present their typical temporal patterns (§3a).

Using the observed CO_2_ growth rate at Mauna Loa as a representation of global NEE, Wang *et al.* [12] showed a close relationship of NEE variations and variations in tropical air temperature (*T*). In order (1) to take into account that the link between climate and NEE acts on local (not global) scales and that NEE–*T* relationships may depend on geographical location and season, and (2) to make systematic use of the additional information in multiple atmospheric CO_2_ records as discussed above, Rödenbeck *et al.* [13] extended such statistical analyses of driving variables for NEE variations by combining a spatially and seasonally resolved linear regression between interannual NEE and *T* variations with an atmospheric inversion. In that study, we found that this ‘NEE–*T* inversion’ captures a large fraction of the interannual NEE variations as seen by a ‘standard inversion’ having explicit interannual degrees of freedom, for both tropical and northern extratropical NEE. Temperature acts as a proxy of climate variations here, representing both direct temperature effects and effects of covarying climate variables such as moisture and incoming radiation (see discussion in [13]). In this study, we extend the NEE–*T* inversion to the 1957–2017 period, which allows transferral of information about ENSO variability from the more data-rich recent decades to the data-sparse 1960s and 1970s (§3b).

Over the 61-year period 1957–2017, however, carbon cycle responses to climatic variations may concievably have been slowly changing due to rising atmospheric CO_2_ (via CO_2_ fertilization and/or changing water use efficiency), changes in vegetation greenness or density, species composition and other factors. Wang *et al.* [14] reported a twofold increase in the sensitivity of the CO_2_ growth rate anomalies at Mauna Loa and the South Pole to anomalies in the tropical annual mean temperature between the 1960s and the 2000s. By contrast, Chylek *et al.* [15] found no significant trend in the response of the Mauna Loa CO_2_ growth rate to the temperature variations during all individual El Niño events since 1960. Here, we re-assess decadal changes in the interannual climate sensitivity of NEE using the multi-station inversions (§3c).

Finally, we present typical spatial patterns of the NEE anomalies during El Niño events, with particular attention to the 2015/2016 El Niño event (§3d).

## 2. Method

### (a) The standard inversion

We estimated spatio-temporal variations of NEE from long-term atmospheric CO_2_ measurements at a set of sites, using an inversion of atmospheric transport (Jena CarboScope system, update of [10,16], see http://www.BGC-Jena.mpg.de/CarboScope/). We performed several inversion runs, listed in table 1. Runs labelled ‘standard inversion’ are essentially identical to the default CarboScope products (v4.2), except that the ocean fluxes are prescribed. The set-up used here is similar to that described in Rödenbeck *et al.* [13], except for the following differences related to the longer analysis period 1957–2017:

**Table 1.** Inversion runs used in this study.

kind of inversion	station set	number of atm. stations	specific feature as of §2(c) (if any)	period of validity	Jena CarboScope run ID
standard	s57	2		1957–2017	s57pt5_v4.2
standard	s57X	7		1957–2017	s57Xpt5_v4.2
standard	s85	21		1985–2017	s85pt5_v4.2
standard	s04	56		2004–2017	s04pt5_v4.2
NEE–*T*	s57X	7		1957–2017	s57Xpt5NEET_v4.2
NEE–*T*	sEXT	87		1957–2017	sEXTpt5NEET_v4.2
NEE–*T*	s57X	7	variable *γ*_NEE–*T*_	1957–2017	s57Xpt5NEET_VarSens_v4.2
NEE–*T*	sEXT	87	no El Niño data	1957–2017	sEXTpt5NEET_NN_v4.2

*Calculation period:* All inversion runs were done over the period 1955–2018 (see table 1 for the usable analysis periods excluding spin-up, spin-down and periods with incomplete data coverage).

**Figure 1.**
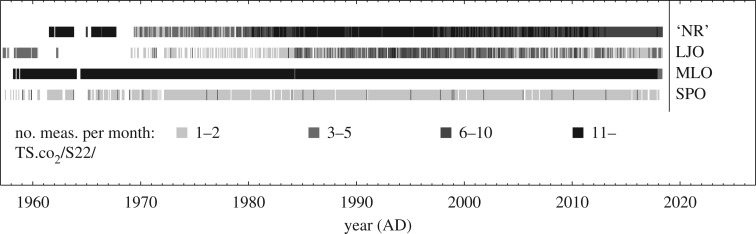
Number of data points available in each month from the records used in the ‘quasi-homogeneous’ station set s57X (ordered by latitude). The ‘northern record’ (NR) is a combined record from several stations (see appendix).

*Station sets:* We performed several runs using different sets of atmospheric stations (table 1). The set s85 consists of 21 stations that are available at least since 1985 but do not all cover the analysis period 1957–2017 targeted here. Only Mauna Loa (MLO, *in situ* data by the Scripps Institution of Oceanography (SIO) [17]) and the South Pole (SPO, flask data by SIO) offer an almost complete coverage (figure 1), and are used together as set s57. In set s57X, we augment these two station records by two records from further north (La Jolla, California (LJO, weekly minima of *in situ* data till 1962 and flask data from 1970, both by SIO) and a combined ‘northern record’ (NR)), as motivated and described in the appendix. It forms a ‘quasi-homogeneous’ dataset that can constrain interannual NEE variations over the full period at least in a coarse latitudinal resolution.

*Prescribed ocean flux:* As the ocean flux product based on *p*CO_2_ data (as used in [13]) is only available from the early 1980s, we used instead the 1957–2017 interannual sea–air CO_2_ flux variations simulated by the PlankTOM5 biogeochemical process model (update of [18]) forced by daily wind, precipitation and air temperature fields from the NCEP reanalysis [19]. The PlankTOM5 model simulates tropical ocean CO_2_ flux variations similar to the *p*CO_2_-based product in the overlapping period (in particular, the simulated amplitude is almost as large as the data-based one, unlike most other ocean process models simulating smaller variations). From the simulations, we only used the interannual anomalies (including the flux trend), by subtracting the 1992–2016 mean and mean seasonal cycle and adding instead the corresponding mean and mean seasonal cycle of the *p*CO_2_ data-based product oc_v1.6 (update of [20]).

### (b) The net ecosystem exchange–*T* inversion

While the standard inversion directly estimates the interannual variations of NEE from the atmospheric CO_2_ signals, the NEE–*T* inversion instead effectively performs a linear regression of interannual NEE anomalies against interannual anomalies of air temperature (see [13] for details). This is done by using spatially and seasonally explicit regression coefficients as adjustable degrees of freedom. These coefficients (*γ*_NEE–*T*_) formally represent the local and season-specific sensitivities of NEE to interannual variations in temperature, but include the sensitivities to other climate variables covarying with temperature. The NEE–*T* inversion is considerably more strongly regularized than the standard inversion, because the regression term involving only 13 degrees of freedom per spatial discretization unit replaces the explicit interannual term with 1320 degrees of freedom per spatial discretization unit.

The NEE–*T* inversion is run either on the ‘quasi-homogeneous’ station set s57X, or on the set sEXT with 87 stations.

### (c) Net ecosystem exchange–*T* inversion runs with specific features

*No El Niño data:* To investigate whether the estimated sensitivity parameters *γ*_NEE–*T*_ only reflect the large NEE and *T* variations during El Niño events but differ for the smaller non-ENSO variations, a specific run was performed where all data points around the seven El Niño events with the largest values of the Multivariate El Niño Index (MEI, [21]) are excluded. In order not to disturb the relative data weight between the seasons, we excluded 2-year periods, namely 1965–1966, 1972–1973, 1982–1983, 1986–1987, 1997–1998, 2009–2010 and 2015–2016. To compensate for the lower number of data (14 missing years out of 61 years) exerting a weaker constraint, the *a priori* uncertainties of all degrees of freedom were increased in the ratio 61/(61 − 14).

*Variable* *γ*_NEE–*T*_: By default, the sensitivity parameters *γ*_NEE–*T*_ of the NEE–*T* inversion are identical in every year of the calculation. To investigate possible long-term changes in the climate sensitivity, we ran the NEE–*T* inversion also with separate independent *γ*_NEE–*T*_ parameters for 20-year windows starting at 1957, 1967, 1977, 1987 and 1997, respectively (actually done through only two runs, one with independent *γ*_NEE–*T*_ parameters for the consecutive intervals 1957–1976, 1977–1996 and 1997–2016, and the other one for 1967–1986 and 1987–2006). As each of the 20-year windows is only constrained by a third of the data, we increased the *a priori* uncertainties of *γ*_NEE–*T*_ by 61/20 for compensation. Like in the default set-up, NEE variations outside these intervals are represented by explicit degrees of freedom as in the standard inversion [13].

### (d) Postprocessing

All inversions give spatio-temporal CO_2_ flux fields nominally on a daily and pixel-scale resolution. Here, we only consider the interannual variations of the land flux (NEE), obtained by applying both running yearly averages (which also remove the seasonal cycle) and a Gaussian spectral filter removing variations faster than about three months. Together, these two filters leave NEE variations on time scales of about 15 months or slower.

For showing time series, we integrated the interannual NEE variations over three regions: globally, over the northern extratropics (90° N–25° N), and over the tropics (taken as 25° N–90° S; the contribution of land areas south of 25° S is very small).

## 3. Results

### (a) What do the longest available atmospheric CO_2_ records say about El Niño Southern Oscillation-related variability of net ecosystem exchange?

**Figure 2.**
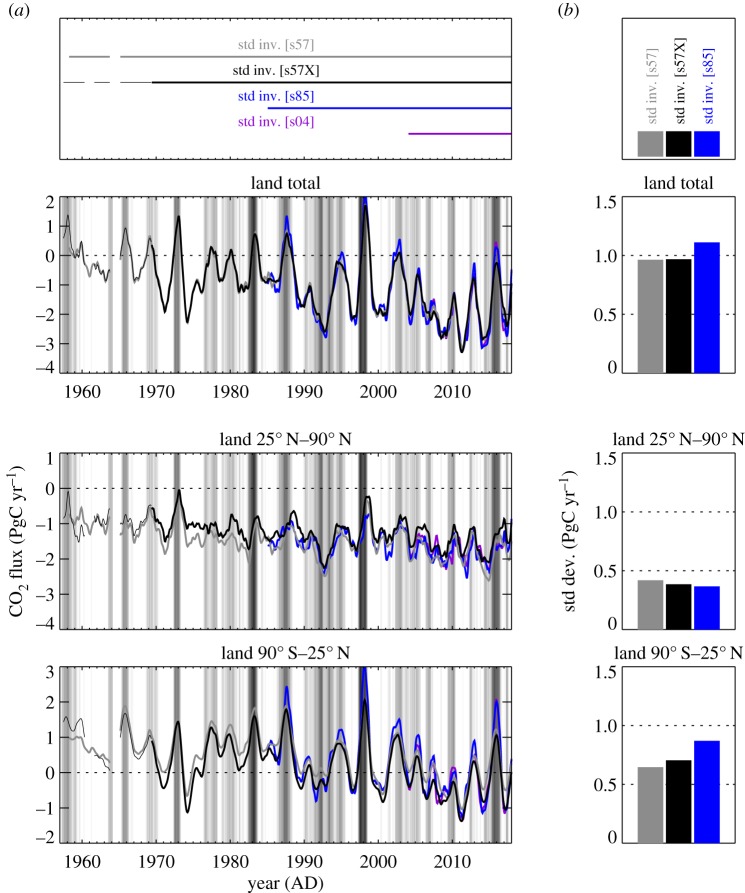
(*a*) Interannual variations of NEE from standard inversions using set s57 (two stations), s57X (seven stations), s85 (21 stations) or s04 (56 atmospheric stations, see table 1). NEE as been filtered for interannual variations, and integrated over all land (top) and latitude bands (middle and bottom). The background shading indicates the Multivariate El Niño Index (MEI) by Wolter & Timlin [21]. (*b*) Temporal standard deviations over 1985–2017.

Figure 2 shows interannual variations (IAV) of NEE, estimated by the standard inversion using differently large sets of atmospheric CO_2_ records (s57, s57X, s85 or s04, see §2a and table 1). The estimates based on set s57 consisting of Mauna Loa, Hawaii (MLO) and South Pole (SPO) cover almost the full analysis period 1957–2017 nearly homogeneously (figure 1). Despite only using two stations, they already give almost the same global NEE total (top panel) as the estimates based on set s85 with 21 stations distributed globally (but only available over 1985–2017), or even the estimates based on set s04 with 56 stations (2004–2017). This agreement in the global total is possible because the atmosphere is essentially mixed within the 15-month time scale shown. Compared with the s85 inversion, however, the s57 inversion attributes too much of this variability to the northern extratropics (middle panel), because it has no station north of MLO (19.53° N) that would contradict such a northward spread of the signal. With the station set s57X where the combined ‘northern record’ (‘NR’, defined and justified in the appendix) and La Jolla Pier (LJO, 32.87° N) are added, the distribution of the variability between the latitude bands already gets closer to that of the s85 run (figure 2*b*, bars). (Run s04 with even more stations than s85—which can be expected to be still more realistic owing to the additional information but which in turn covers an even shorter period (figure 2*a*—differs in several detailed features, but essentially confirms the IAV amplitudes of the s85 run in the two latitude bands.)

**Figure 3.**
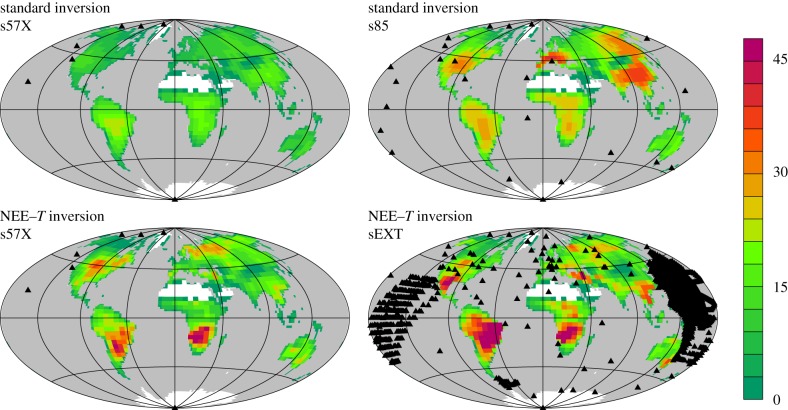
Amplitudes of interannual NEE variations (gC m^−2^ yr^−1^), calculated as temporal standard deviations over 1985–2017. Black triangles indicate the sampling locations of the atmospheric data used in the individual inversions.

Though the s57X inversion still has too small interannual variations in the tropics, their actual temporal course in the two latitude bands does not differ much from that of the s85 inversion (figure 2*a*). Further, maps of interannual amplitudes (figure 3) reveal that the s57X inversion, despite having a station coverage far from global, assigns interannual variability to all continents (top left map), in spatial proportions not too different from those of the s85 inversion (except Europe, top right map). We conclude that the smaller variability of the s57X inversion arises from the relatively low Bayesian weight of the data constraint exerted by the few stations against the dampening *a priori* constraint, rather than fundamentally missing information. Tests with increased data weight (not shown) confirm this view. Keeping the deficiency in its amplitude in mind, we therefore take the inversion with station set s57X (henceforth referred to as ‘quasi-homogeneous’ set) as default estimate, as it offers the best available compromise between the necessities to use temporally homogeneous data constraints over 1957–2017 but also to include stations north of MLO as shown above.

**Figure 4.**
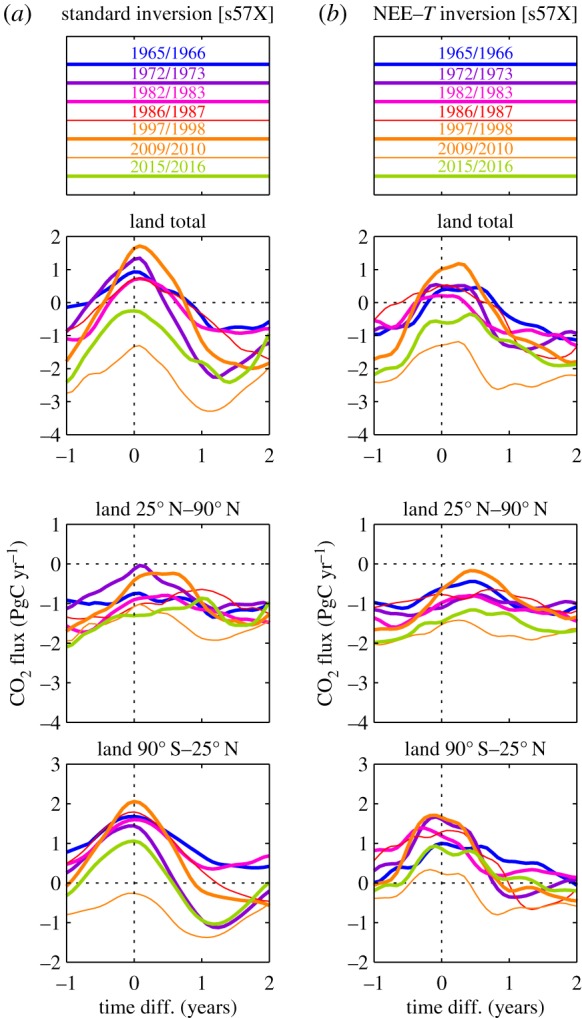
Interannual NEE time series sections around the seven El Niño events with the highest MEI values [21] within 1957–2017: the origin of the time difference axis denotes the beginning of November 1965, January 1973, April 1983, August 1987, March 1998, March 2010 or January 2016, respectively. NEE has been estimated by the standard inversion (*a*) or by the NEE–*T* inversion (*b*), both using the ‘quasi-homogeneous’ station set s57X.

Like the recent decades, the early decades also follow the pattern of enhanced CO_2_ emissions in the tropics during El Niño events, and of enhanced CO_2_ emissions in the northern extratropics some months after El Niño events. This pattern is even better seen on the magnified time axis in figure 4*a*.

### (b) How is El Niño Southern Oscillation variability of net ecosystem exchange linked to climate variability?

**Figure 5.**
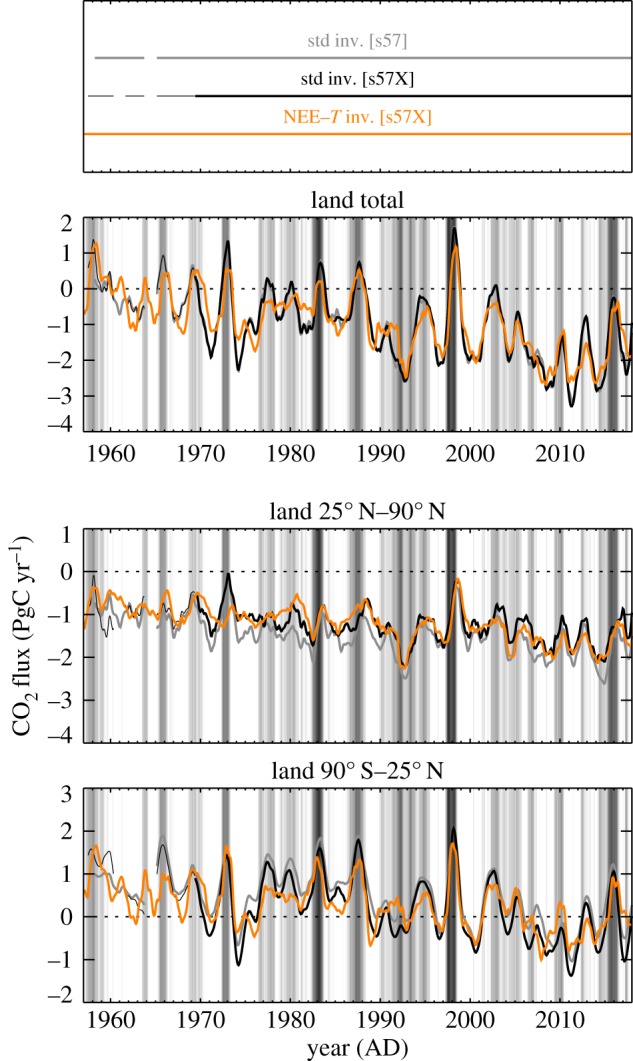
Interannual variations of NEE from standard and NEE–*T* inversions (IAV filtered/integrated as in figure 2). Black and orange runs use the ‘quasi-homogeneous’ station set s57X.

The NEE–*T* inversion, whose NEE variability originates entirely from temperature variability (§2b), aligns for almost all peaks and troughs with the interannual NEE variability inferred from the ‘quasi-homogeneous’ set s57X by the standard inversion having explicit interannual degrees of freedom (figure 5). The good temporal alignment is confirmed by the magnified time axis in figure 4 (right compared with left). It reveals, however, a slight double-peak structure of the tropical El Niño peaks from the NEE–*T* inversion (bottom right) not present in the standard inversion (bottom left). This double-peak structure seems to arise because various subregions of the 90° S–25° N band have their peaks at slightly different times. The information about this different timing is added to the NEE–*T* inversion by the temperature field, while the standard inversion based on very few stations would not be able to resolve such geographical differences. This is also seen in the more heterogeneous structure of amplitudes of the NEE–*T* inversion (figure 3, bottom left), while the standard inversion produces a rather smooth NEE field (figure 3, top left).

**Figure 6.**
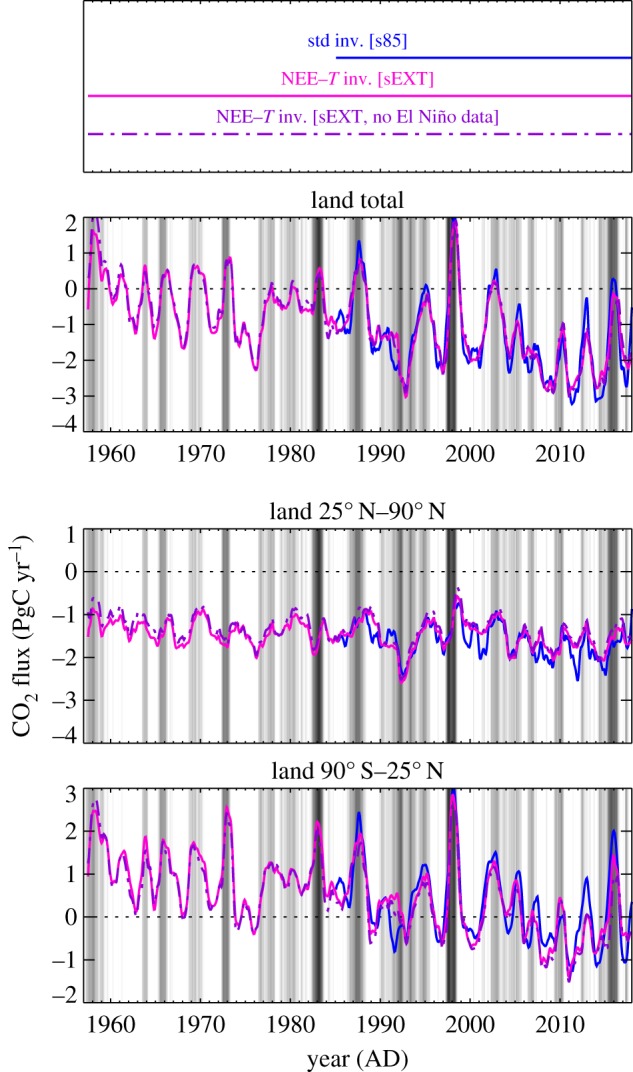
As figure 5, but using station set s85 in the standard inversion and sEXT in the NEE–*T* inversion. In addition, the test run discarding all data points during 2-year intervals around the seven MEI-strongest El Niño events is shown.

As also found for the standard inversion (§3a), the IAV amplitude of NEE from the NEE–*T* inversion increases when more measurement stations are used (figure 6 compared with figure 5). Since the NEE–*T* inversion can also cope with records not spanning the full analysis period, we run it with station set sEXT comprising 87 stations. Even though the interannual NEE variations themselves originate from temperature variations in the NEE–*T* inversion, a larger set of atmospheric stations should help to determine the spatial and seasonal pattern of the sensitivities *γ*_NEE–*T*_ more correctly (figure 3, bottom right). We therefore expect the NEE–*T* inversion with station set sEXT to provide the best spatial resolution among the estimates presented here, and will therefore use it in §3d below.

Are the largest El Niño events dominating the inferred relation between NEE variations and climate variations? We tested this by a specific run of the NEE–*T* inversion discarding the data from 2-year periods around the seven El Niño events with the largest MEI [21]) within 1957–2017 (§2c). This changes the resulting NEE variations only very little (figure 6). Even the large peaks—for which no direct data information is available in the test run—are essentially correctly predicted based on the knowledge ‘learnt’ from the smaller El Niño events and the La Niña variability.

### (c) Did the ‘interannual climate sensitivity’ change over the 61-year analysis period 1957–2017?

In the NEE–*T* inversion runs presented so far, the estimated sensitivity parameters *γ*_NEE–*T*_ are set up to be temporally constant over all the analysis period 1957–2017. To investigate potential changes in the climate sensitivity, we performed specific test runs of the NEE–*T* inversion with separate independent *γ*_NEE–*T*_ parameters for the 20-year windows starting at 1957, 1967, 1977, 1987 and 1997, respectively (§2c). Indeed, we found changes in the local sensitivities over time. These changes are different in different geographical areas and different seasons, and mostly do not follow monotonic temporal trends (not shown).

In order to see the global effect of these changes, we looked at regression coefficients between the interannual variations in global total NEE and interannual variations in tropical air temperature, calculated over the before-mentioned 20-year windows. For tropical air temperature, we used the temperature field from the NEE–*T* inversions with decadal variations removed, averaged over the 25° N–25° S land areas. Before the regression, both NEE and temperature time series were detrended, and interannually filtered as described in §2d.

**Figure 7.**
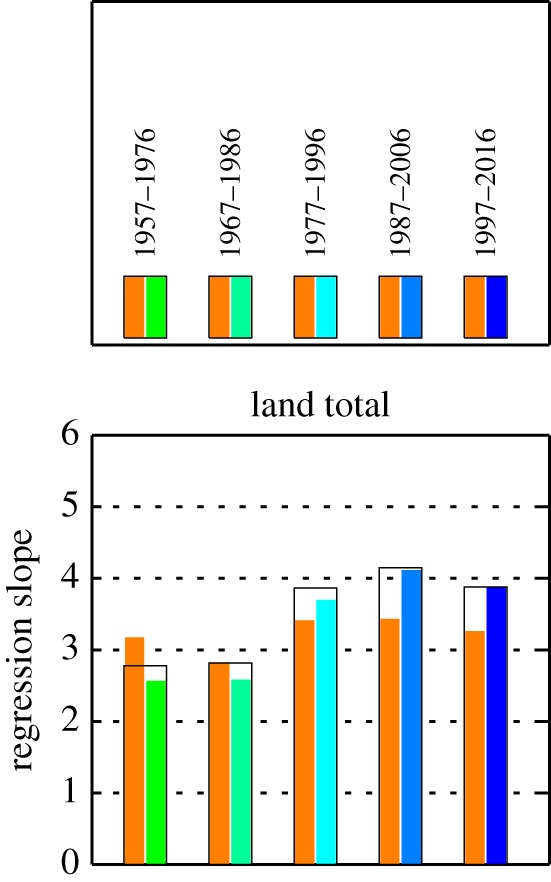
Regression slopes ((PgC yr^−1^) K^−1^) between interannual variations of global terrestrial NEE and of tropical mean air temperature (over 25° N–25° S land regions, without decadal variations as used in the NEE–*T* inversion) for five 20-year windows. NEE has been estimated by the standard inversion (hollow black bars), the NEE–*T* inversion with the same ‘interannual climate sensitivity’ throughout 1957–2017 (as in figure 5, orange bars), and NEE–*T* inversions with independent sensitivities in each of the 20-year windows (green-to-blue bars). All inversions use the ‘quasi-homogeneous’ station set s57X. Before the regression, all time series were interannually filtered, and any linear trends have been removed.

Figure 7 shows the resulting regression slopes, i.e. the apparent global climate sensitivities. Those calculated from the standard inversion (only representing data information, black hollow bars) and from the NEE–*T* inversion with variable *γ*_NEE–*T*_ (green-to-blue bars) agree relatively well with each other, and indeed rise from the 1957–1976 window to the 1987–2006 window. The rise by about 1.5 (1.6) is somewhat lower than the factor of 1.9 ± 0.3 reported by Wang *et al.* [14], possibly also due to the slightly different ways used to calculate the global sensitivity. In the most recent window, 1997–2016 (after the analysis period of [14]), however, the apparent global climate sensitivity drops down again to about the 1.4-fold (1.5-fold) of its 1957–1976 value.

Maybe surprisingly, the 20-year regression slopes from the NEE–*T* inversion with constant *γ*_NEE–*T*_ (orange bars) change over time in a similar pattern, just less strongly (e.g. a 1.2-fold increase from 1967–1986 to 1987–2006 instead of 1.6-fold when *γ*_NEE–*T*_ is variable). Despite limited confidence in the absolute ratios (see below), this indicates that by far not all the changes in sensitivity inferred from the data are actually related to decadal physiological changes, while the remaining changes must just arise from shifts in the geographical areas or seasons that most contribute to the tropical mean temperature: if the dominant areas (or seasons) have lower/higher local (or season-specific) climate sensitivity, the apparent sensitivity calculated from large-scale annual NEE and large-scale annual temperature will be lower/higher as well, even without any actual physiological change.

All runs for figure 7 are based on the ‘quasi-homogeneous’ station set s57X, in order to minimize the influence of decadal changes in the data constraint (§3a). Nevertheless, the sensitivities calculated in the earliest 20-year window might be underestimated because of the somewhat weaker data constraint due to the gaps in the ‘northern’ and LJO records. For the more recent 20-year windows, runs with more stations confirm the decadal pattern of the regression slopes, though all the regression slopes become larger (not shown); larger regression slopes are consistent with the larger amplitudes of interannual variations (figure 6 compared with figure 5). We also note that the exact values of the regression slopes depend to some extent on the filtering applied to the NEE and temperature time series, in particular on the cut-off frequency of the decadal variations being removed.

### (d) Which parts of the Earth's surface are most affected by El Niño Southern Oscillation-related net ecosystem exchange variability?

**Figure 8.**
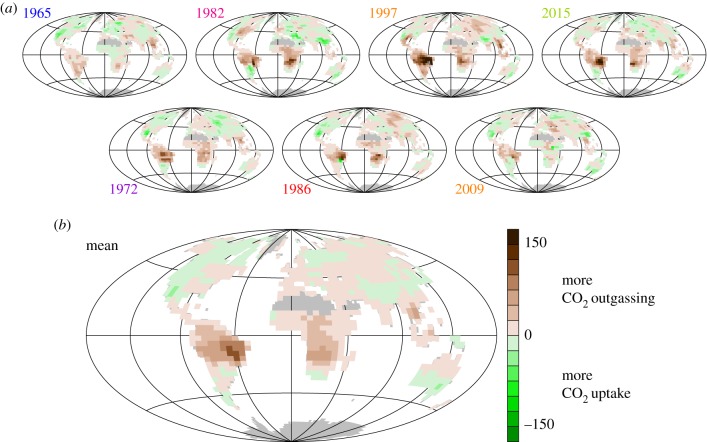
Spatial signature of El Niño: NEE anomalies estimated by the NEE–*T* inversion with station set sEXT (interannual anomalies around the 1957–2017 linear trend, in gC m^−2^ yr^−1^). (*a*) Maps for the seven individual El Niño events with largest MEI [21] (taken at beginning of November 1965, January 1973, April 1983, August 1987, March 1998, March 2010 and January 2016). (*b*) Mean over these events.

Figure 8*a* shows maps of the NEE anomalies for individual large El Niño events. We use here the NEE–*T* inversion with the large sEXT station set as discussed in §3b. Corresponding to the specific climate anomaly patterns of the individual events, the spatial patterns of NEE are different in the details, but the mean over these events (figure 8*b*) reveals systematic responses mainly in the tropics, in particular, South America, tropical and Southern Hemispheric Africa, and south Asia. The 2015/2016 El Niño event focused on in this special issue conforms with the mean spatial pattern, with one of the largest amplitudes. Particularly large responses are estimated in the Amazon basin and in Southern Hemisphere Africa.

## 4. Conclusion

We estimated interannual variations of terrestrial NEE of CO_2_ over the period 1957–2017 from atmospheric CO_2_ measurements. From the few observational records already available in the early part of this period, we formed a ‘quasi-homogeneous’ set as a compromise between avoiding spurious jumps from starting/ending records and nevertheless offering sufficient information to distinguish at least northern extratropical and tropical variability. Consistent with previous findings, the estimates show enhanced CO_2_ outgassing during El Niño events in the tropical band, and enhanced CO_2_ outgassing a few months later also in the northern extratropical band, throughout 1957–2017.

Despite the complexities of the underlying processes, the response of the terrestrial carbon cycle to El Niño climate anomalies is well approximated by a spatially/seasonally resolved linear relationship between NEE anomalies and *T* anomalies taken as a climate proxy. The regression coefficients *γ*_NEE–*T*_, interpreted as ‘interannual climate sensitivity’, not only depend on the largest anomalies, but can also be inferred by excluding the data during the large El Niño events.

The apparent climate sensitivity of global NEE with respect to tropical annual mean air temperature increased from the 1960s and 1970s to the 1990s and early 2000s (though not as strongly as reported by Wang *et al.* [14]), but decreased again afterwards. However, only part of these changes are actually due to changes in the local or season-specific climate sensitivity reflecting physiological or ecosystem processes, while the rest arises from shifts in the location of the dominant climate variability.

## Figures and Tables

**Figure 9.**
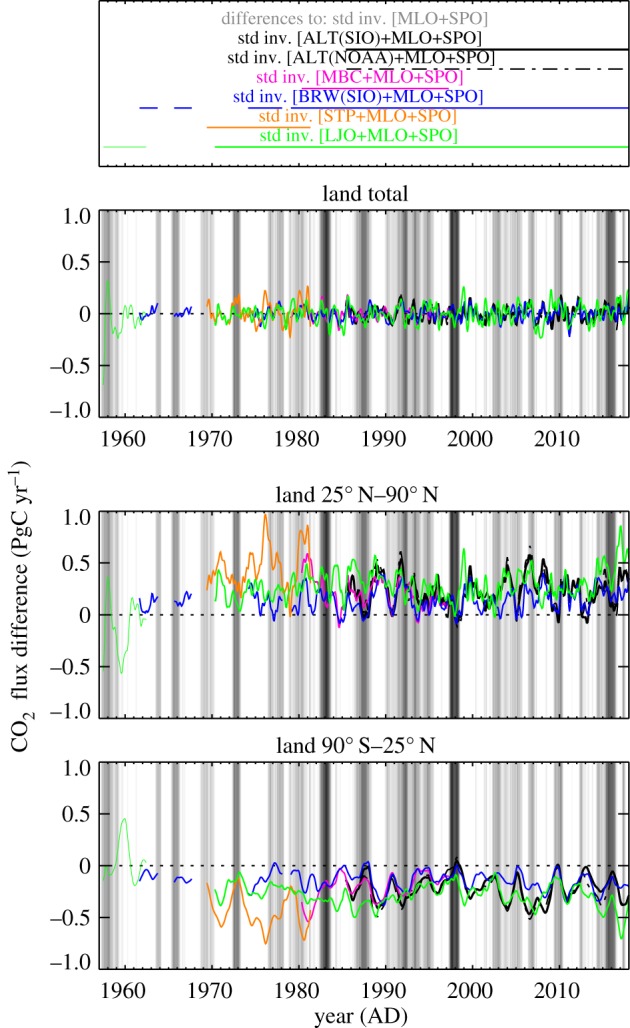
Assessing the signals added to the inversion by various atmospheric CO_2_ records. Differences between the estimated interannual NEE variations from standard inversions using three stations (the test record in addition to MLO and SPO) and a reference standard inversion using two stations (MLO and SPO only). Though all runs were carried out over the same period (1955–2018), the results are only plotted over the time sections covered by all records involved.

## Data accessibility

The inversion results are available for use in collaborative projects from the Jena CarboScope website http://www.BGC-Jena.mpg.de/CarboScope/.

## Appendix: Data used for the ‘northern record’

At the high northern latitudes, none of the available data records spans the entire analysis period 1957–2017. We therefore combined several northern records that provide similar signals to the inversion. The signals were assessed by standard inversions based on the respective record together with Mauna Loa (MLO) and South Pole (SPO). From the results of these inversions, we subtracted fluxes estimated with only MLO and SPO (set s57), in order to see the signal introduced by the test record more clearly. As seen in figure 9, the total land flux (top panel) is not much changed by any record (except in 1957 as discussed below) because it is already relatively well constrained by MLO and SPO (see discussion in §3a). However, the additional records shift fluxes between the northern extratropical and tropical latitude bands (middle and lower panel).

For the ‘northern record’ we use the following stations:

— At Alert (ALT), we use the flask records by SIO [17] and by the National Oceanic and Atmospheric Administration of the USA (NOAA) [22], which are very compatible with each other (figure 9, two black lines).
— Data by NOAA from Mould Bay, Canada (MBC) provide very similar signals to ALT as well and extend its coverage further back in time (magenta).
— The earliest high northern measurements were done after July 1961 by Kelley [23] and SIO at Point Barrow, Alaska (BRW), though the measurements have a gap in 1964 (like SPO) and stopped after 7 years, to be continued from 1971 by SIO. The signal from BRW (blue) is similar in many temporal features to that from ALT, though it causes smaller flux differences for several of ALT’s peaks in the 2000s. Interestingly, a record of *in situ* measurements by NOAA at BRW (not shown) leads to flux differences more similar to ALT. This may be related to the air sampling at 16 m height for the NOAA *in situ* record but only 2 m for the SIO flask record, which may bias the CO_2_ values low in late summer when the tundra vegetation around BRW is active [24].
— In order to partially fill the long gap of BRW between 1967 and 1974, we further added Ocean Station P (STP, 50° N). It is located more away from the other stations, all in Alaska, but is the closest one available at that time. STP causes somewhat larger deviations than the other records (orange), but we nevertheless use it as a ‘northern station’ because, in the period of overlap, MBC has a similar feature, which is larger than MBC’s later variability.

We combined the data points of all of these records into a ‘northern record’ to be used like a single station in the inversions. Every data point is still used at its original location and time, but owing to the data density weighting implemented in the Jena CarboScope algorithm [16, pp. 8–11], the combined record roughly has the same weight in the cost function throughout time (except for the remaining gaps in the 1950s and 1960s), reducing the impact of the changing number of stations contributing to the ‘northern record’ (as opposed to using the same stations individually).

In addition to the stations used for the ‘northern record’, figure 9 also shows the signal from La Jolla Pier (LJO, 32.87° N, green), used as a separate station in the ‘quasi-homogeneous’ set s57X. Though located further south, it agrees with the ENSO-related features from ALT remarkably closely. In the early 1970s, the temporal course inferred from LJO is similar to that from STP, except for STP’s larger amplitude. There is a period of low variability in the late 1970s and early 1980s, not inferred by the other stations, which we cannot presently explain. Nevertheless, we take the existing similarities as an indication that LJO and the ‘northern record’ share at least part of the signal and therefore complement each other during their gaps in the earliest decade.

The earliest LJO data create flux differences larger than any of the other stations in figure 9. The shift in the land total in 1957 may be related to the fact that MLO data only start in 1958, such that the two-stations s57 inversion is badly constrained in 1957 and therefore easily changed by the additional station. We assume that LJO can take MLO’s role as a Northern Hemispheric station in the station set s57X in 1957, and that we therefore can use the results right from 1957. There is a second peculiar feature from LJO in 1959–1960, with more negative fluxes in the northern extratropics and correspondingly larger fluxes in the tropics, even though there is no El Niño event. We do not yet have an explanation and assume this feature to be an artefact. Doubts also come from the fact that the s57 standard inversion (without LJO) fits better to the NEE–*T* inversion during 1959–1960 than the s57X inversion including LJO (figure 5, middle panel).

We note here that we sampled the modelled mole fractions of LJO at a northwest-shifted location (40° N, 126° W) and 2 days in advance of the measurements. This takes into account that—owing to the flask sampling rules applied at LJO—the measurements represent air arriving at LJO from more northern areas through a strong and narrow jet parallel to the coast. As the transport model is too coarse to resolve this local circulation feature, the inversion would otherwise misinterpret LJO data, leading to additional large variability in the estimated northern extratropical CO_2_ flux.
